# Facilitation of the inhibitory transmission by gastrin-releasing peptide in the anterior cingulate cortex

**DOI:** 10.1186/1744-8069-6-52

**Published:** 2010-09-13

**Authors:** XiaoYan Cao, Valentina Mercaldo, Pingyang Li, Long-Jun Wu, Min Zhuo

**Affiliations:** 1 Department of Physiology, Faculty of Medicine, University of Toronto, 1 King's College Circle, Toronto, Ontario M5S 1A8, Canada; 2 Department of Brain and Cognitive Sciences, College of Natural Sciences, Seoul National University, Seoul 151-746, Korea

## Abstract

Gastrin-releasing peptide (GRP) has been proposed as a peptidergic molecule for behavioral fear and itching. Immunohistochemistry and in situ hybridization studies have shown that GRP and GRP receptor are widely distributed in forebrain areas. Less information is available for the functional action for GRP in the prefrontal cortex including the anterior cingulate cortex (ACC). Here we used whole-cell patch-clamp recording technique to study the modulation of synaptic transmission by GRP in the ACC. We found that GRP increased the frequency of sIPSCs recorded while had no significant effect on sEPSCs in ACC pyramidal neurons. The facilitatory effect of GRP on sIPSCs was blocked by the GRP receptor antagonist, RC3095. In the presence of TTX, however, GRP had no effect on the mIPSCs. Therefore, activation of GRP receptor may facilitate the excitation of the interneurons and enhanced spontaneous GABAergic, but not glutamatergic neurotransmission. Similar results on GRP modulation of GABAergic transmission were observed in the insular cortex and amygdala, suggesting a general possible effect of GRP on cortical inhibitory transmission. Our results suggest that GRP receptor is an important regulator of inhibitory circuits in forebrain areas.

## Introduction

Gastrin-releasing peptide (GRP) is a mammalian analogue of bombesin (BB), a 14 amino acid-containing peptide first isolated from the skin of the frog *Bombina bombina *[1,2]. Anatomic studies have shown that GRP and its receptors are widely distributed in the central nervous system, in addition to the gastrointestinal (GI) tract [2-10]. GRP has been implicated in many physiological and pathological conditions such as the regulation of the circadian rhythm, exocrine and endocrine secretions, smooth muscle contraction, inflammation, feeding, fear and behavioral itch [11-17].

Recent studies on GRP in sensory systems have triggered new interests on GRP. At the level of the spinal cord, it has been reported that GRP may serve as a selective signaling transmitters for itching sensation [14,15]. In the amygdala, it has been reported that GRP may contribute to regulation of neuronal excitability, and contribute to behavioral fear [18]. Although it has been known that GRP is distributed in cortical areas, less is known about the possible modulatory effects of GRP on cortical circuits. The anterior cingulate cortex (ACC)**,**a key structure of the prefrontal cortex, plays an established role in learning and memory, drug addiction, and chronic pain [19-22]. In the present study, we have investigated the effects of GRP on both excitatory and inhibitory transmission in the ACC. Our results show that the GRP selectively facilitate GABAergic but not glutamatergic neurotransmission. The facilitation may result from the GRP-induced inward current and firing of GABAergic interneurons in the ACC.

## Methods

### Animals

Adult male C57BL/6 mice were purchased from Charles River (6-10 weeks old). All mice were maintained on a 12 h light/dark cycle with food and water provided *ad libitum*. All protocols used were approved by The Animal Care and use Committee at the University of Toronto and conform to NIH guidelines.

### Whole-cell Patch Clamp Recordings

Adult male mice were anesthetized with 1-2% halothane and decapitated. Coronal slices (300 μm) containing the ACC, amygdala or insular cortex will be prepared using routine methods used in our laboratory [23,24]. Slices were then transferred to a submerged recovery chamber with oxygenated (95% O_2 _and 5% CO_2_) ACSF at room temperature. After a one-hour recovery period, slices were placed in a recording chamber on the stage of an Axioskop 2FS microscope (Zeiss) equipped with infrared DIC optics for visually-guided whole cell patch clamp recordings. Pyramidal neurons or interneurons in Layer II-III in the ACC were recorded with an Axon 200B amplifier (Molecular device, Union city, CA). Recording electrodes (2-5 M) contained an internal solution composed of (in mM): Kgluconate, 120; NaCl, 5; MgCl_2 _1; EGTA, 0.5; Mg-ATP, 2; Na_3_GTP, 0.1; HEPES, 10; pH 7.2; 280-300 mOsmol. The membrane potentials were held at -70 mV throughout all experiments. When recording GABA_A _receptor-mediated currents, K-gluconate was replaced by Cs-MeSO_3 _and a holding potential of 10 mV. Spontaneous EPSCs were recorded in the presence of GABA_A _receptor antagonist, picrotoxin (100 μM) and spontaneous IPSCs were recorded in the presence of a NMDA receptor antagonist, AP5 (100 μM) and a non-NMDA receptor antagonist, CNQX (20 μM). GRP and its receptor antagonist RC3095 were purchased from Sigma. To examine the mIPSCs, TTX (1 μM) was bath-applied in the perfusion solution. The sIPSCs/mIPSCs were analyzed with the Mini Analysis Program v5.2.4 (Synaptosoft Inc., Decatur, GA). Access resistance was 15-30MO and monitored throughout the experiments. Data were discarded if access resistance changed > 15% during an experiment. Signals were filtered at 1 kHz, digitized at 10 kHz.

### Passive and Active Membrane Properties

Off-line analysis was performed using Clampfit version 9 (Axon Instruments). Resting membrane potential (RMP) was the low-pass readout of the electrode amplifier and was not corrected for liquid junction potential (~12 mV) after terminating the recording. The membrane potential was measured immediately after establishing the whole-cell configuration. Only neurons that had a resting membrane potential more negative than -60 mV were further investigated. Conductance was determined from the linear slope (between -60 mV to -80 mV) of the current-voltage (I-V; V_hold _= -70 mV) relationships. Action potentials (APs) were detected in response to suprathreshold current injections from a holding potential around -70 mV. Depolarizing currents of 5~200 pA (400-ms duration) were delivered in increments of 5 pA until an AP was evoked. The rheobase was defined as the minimum current required to evoke an action potential. The AP voltage threshold (Vthres_hold_) was defined as the first point on the rising phase of the spike at which the change in voltage exceeded 50 mV/ms. The spike amplitude was quantified as the difference between the Vthres_hold _and the peak voltage. The spike width was measured at 1/2 of the total spike amplitude (measured from the Vthres_hold _level). The amplitude of the afterhyperpolarization (AHP) was estimated as the difference between the Vthres_hold _and the peak of AHP.

### Immunohistochemistry

Mice were deeply anesthetized with halothane and perfused transcardially with 50-100 ml saline followed by 150-500 ml of cold 0.1 M phosphate buffer (PB) containing 4% paraformaldehyde. Brains were removed and post-fixed in 4% paraformaldehyde/PBS and then will be placed in 30% sucrose in 0.2 M PBS, embedded in the OCT compound and frozen. Coronal sections (30 μM thickness) were cut using a cryostat. For dual fluorescent immunohistochemistry, sections were incubated overnight with anti-GRPR (1:50; rabbit polycolonal, Santa Cruz Biotecnology) and anti-GAD67 (1:300; mouse monoclonal, Chemicon) antibodies at 4°C. Sections were then be washed 3 times with PBS 0.1 M and incubated for 2 hours with anti Mouse-FITC and rabbit-rhodamine conjugated secondary antibodies (1:200; Chemicon). Images of the ACC areas of at 0.7-μm intervals with 20× lens were obtained with Bio-Rad Laboratories MRC 1000 laser-scanning confocal fluorescent imaging system.

### Data Analysis

Results were expressed as mean ± standard error of the mean (S. E. M.). Statistical comparisons were performed with the use of one- or two-way analysis of variance (ANOVA) with the post-hoc Scheffe F-test in immunocytochemical experiments. Analysis of mIPSCs/sEPSCs was performed with cumulative probability plots and was compared using the Kolmogorov-Smirnov (K-S) test for significant differences. In all cases, P < 0.05 was considered statistically significant.

## Results

### The expression of GRP and GRP receptors in the ACC

**Figure 1:**
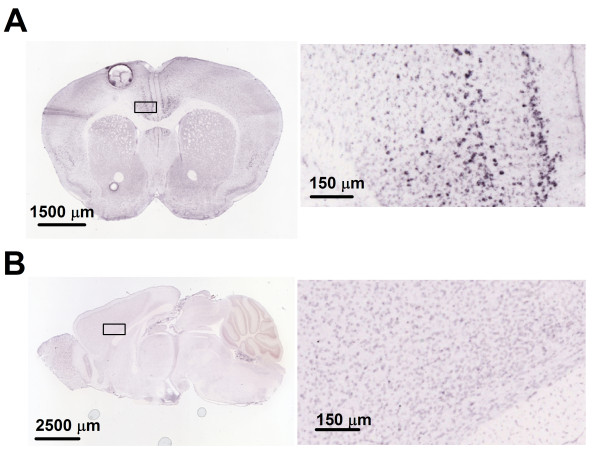
**Expression of the *GRP *and *GRPR *in the ACC (from the Allen Mouse Brain Atlas)**. **(A) **In situ hybridization on a coronal section of mouse brain, showing the expression of the *Grp *gene in the anterior cingulate cortex (ACC) of wild-type mice (rectangled area http://mouse.brain-map.org/viewImage.do?imageId=79611383). **(B) **In situ hybridization on a sagittal section of mouse brain, showing expression of the *Grp *receptor gene in ACC of wild-type mice (rectangled area http://mouse.brain-map.org/viewImage.do?imageId=73493204).

**Figure 2:**
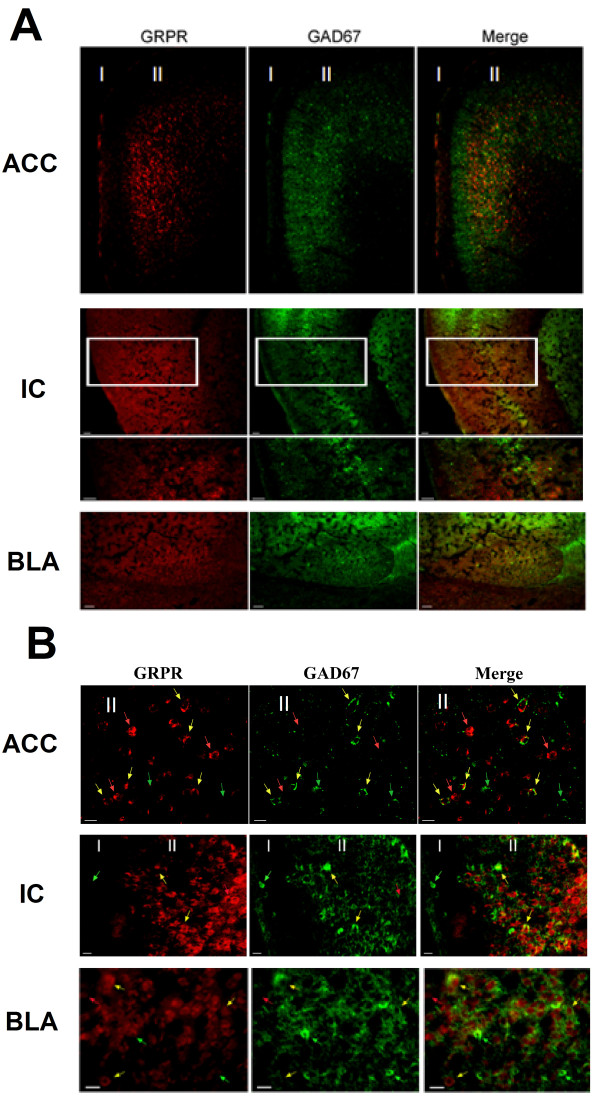
**GABA immunoreactivity in the GRPR neurons**. Double-immunostaining was performed using anti-GRP-R antibody (in red, left column) and an antibody specific for GABAergic neurons (anti-GAD67 antibody, in green center column) in the anterior cingulate cortex (ACC), basolateral amygdale (BLA) and insular cortex (IC). A subpopulation of GRP-R immunoreactivity was detected in GABAergic neurons (arrows), merged in right column. **(A) **Upper panels show images from low-powered microscopy. Scale bar = 10 μm. **(B) **Lower panels show parts of the same sections at higher magnification. Scale bar = 50 μm.

To understand if GRP may be distributed in mouse ACC, we took the advantage of online brain mapping data base (the Allen Mouse Brain Atlas). We found that in adult mouse brains the *Grp*gene is highly enriched in the ACC (Figure 1A); *http://www.alleninstitute.org*), including the regions of Cg1 and Cg2. Consistent with *Grp *distribution in the ACC, the Grp receptors (GRPR) mRNA are also highly expressed in the ACC (Figure 1B); taken from *the Allen Mouse Brain Atlas*). To confirm the distribution of GRP receptor in the ACC, we performed double-label immunohistochemistry using specific antibodies for GRP receptor and GABAergic neurons (see(Figure 2A and 2B). In addition to the ACC, we have also examined the regions of the insular cortex (IC) and basal lateral amygdala (BLA). We found that GRP receptor was highly expressed in these areas. Particularly, GRP receptor was co-localized with GAD67, a marker for GABA in some neurons, suggesting GRP receptor is expressed in a subpopulation of GABAergic interneurons. These results indicate that GRP may play modulatory roles in these brain areas.

### Morphological and electrophysiological properties of interneurons and pyramidal neurons in the ACC

**Figure 3:**
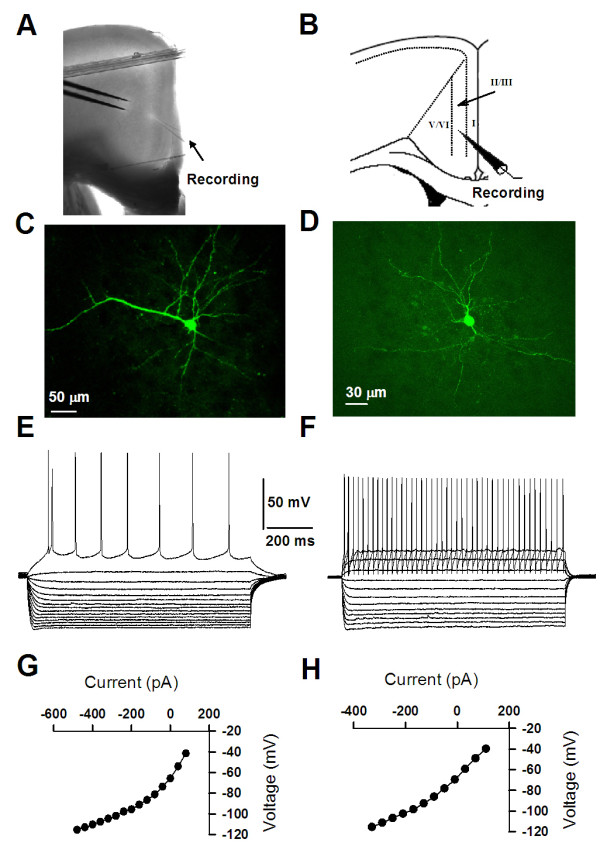
**Morphological and electrophysiological properties of interneurons and pyramidal neurons in the ACC**. **(A) **Representative coronal section showing the placement of a whole-cell patch recording in a cingulate slice. **(B) **Diagram representation of the location of the recorded neurons in layer II/III. **(C and D) **Photomicrograph of a representative biocytin--labeled layer II/III ACC pyramidal neuron (**C**) interneuron (**D**) as visualized with confocal laser scanning microscopy. **(E) **Pyramidal neurons showed different firing properties from those observed in interneurons after current injection (see F). **(F) **Interneurons were identified by their firing properties. When injected with current step (100 pA within 400 ms), interneurons showed fast spiking properties. **(G and H) **current-voltage relationship constructed from values taken at the end of pulses (dots in *E *and *F*).

Since GRP receptor is expressed in many neurons in the ACC, we wanted to know whether it may function differently in different types of neurons, i.e pyramidal neurons and inhibitory interneurons. Next we performed whole-cell patch-clamp experiments to examine the effects of GRP on ACC neurons. As previously reported, we distinguished local inhibitory neurons and pyramidal neurons morphologically and electrophysiologically [23,25]. In some experiments, we also performed whole-cell patch clamp recording combined with biocytin labeling in ACC neurons (see(Figure 3 for examples). Typical layer II/III pyramidal cells had a prominent apical dendrite, which ascended toward the superficial layers, while their basal dendrites were mainly located within the same layer as the soma (Figure 3C). Somata of interneurons were usually ovoid in shape and the soma size was smaller than that of pyramidal cells. In contrast to the pyramidal cells, interneuron was lack of the apical dendrite and displayed 2-5 primary dendrites extending in all directions ((Figure 3D).

Interneurons and pyramidal cells were found to exhibit significantly different input resistances and resting membrane potentials (see Table 1). In response to a continuous depolarizing conditions, pyramidal cells usually fired APs with an rapid initial phase followed by a slow gradual decline, a phenomenon called "spike frequency adaptation"(Figure 3E), whereas interneurons fired a regular train of APs (Figure 3F). We also compared the voltage-current relationship (I-V) of pyramidal neurons and interneurons. The I-V for pyramidal neurons was linear at membrane potentials between -65 and -90 mV, with inward rectification between -90 mV and -120 mV (Figure 3G), while for interneurons was linear between -40 mV and -80mV and showed a slight inward rectification between -100 mV and -120 mV (Figure 3H). Moreover, we found that interneurons exhibited a less Rheobase current (interneurons: 56.8 ± 11.8 pA, *n *= 11; pyramidal neurons: 95.7 ± 7.3 pA, *n *= 11, *p <*0.01), a lower action potential amplitude (interneurons: 88.2 ± 2.6 mV, *n *= 11; pyramidal neurons: 93.8 ± 0.8 mV, *n *= 11, *p <*0.01), a narrower action potential half-width (interneurons: 1.00 ± 0.05 ms, *n *= 11; pyramidal neurons: 1.50 ± 0.03 ms, *n *= 11, *p <*0.001) and a bigger after-hyperpolarization (interneurons: -24.8 ± 2.5 mV, *n *= 11; pyramidal neurons: -6.9 ± 0.3 mV, *n *= 11, *p <*0.001) compared to the pyramidal cells (see Table 1).

**Table 1:** Basic electrophysiological properties of ACC pyramidal and inhibitory neurons

	Pyramidal Neuron	Interneuron	Significant Difference
Number of neurons tested	33	18	

Resting membrane potential, mV	-73.0 ± 1.0	-61.4 ± 1.9	P < 0.001

Input resistance, M Ω	124.3 ± 6.3	332.4 ± 48.1	P < 0.001

Rheobase current, pA	95.7 ± 7.3	56.8 ± 11.8	P < 0.01

Action potential threshold, mV	-35.4 ± 0.4	-43.0 ± 0.9	P < 0.001

Action potential amplitude, mV	93.8 ± 0.8	88.2 ± 2.6	P < 0.01

Action potential half-width, ms	1.50 ± 0.03	1.00 ± 0.05	P < 0.001

Amplitude of after-hyperpolarization, ms	-6.9 ± 0.3	-24.8 ± 2.5	P < 0.001

Values are Mean ± SEM. Significant differences were obtained by the two-tailed, unpaired student t-test.

### GRP induced inward currents in interneurons but small or undetectable currents in pyramidal neurons

To examine the function of GRP receptor in the ACC, GRP was applied through bath solution and the responses of pyramidal neurons and interneurons were recorded. At holding potential of -70 mV a short application of GRP (300 nM) induced a slowly developing inward current (peak 16.9 ± 1.8 pA) that recovered slowly over 10 min (n = 8/10 interneurons). However, in pyramidal neurons there was undetectable currents upon GRP application (300 nM, n = 7). In the presence of GRP receptor antagonist, RC3095 (3 μM), the effect of GRP induced inward current in interneurons (n = 7) was completely blocked (data not shown). These results indicate that GRP selectively activated interneuronal GRP receptor in the ACC.

### Activation of GRP receptors enhances spontaneous GABAergic, but not glutamatergic transmission

**Figure 4:**
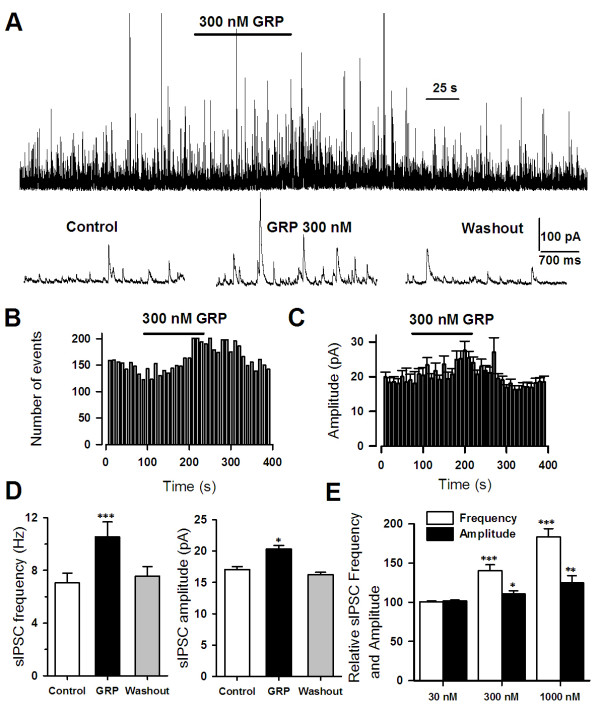
**Activation of GRP receptor by GRP reversibly increased sIPSCs in pyramidal neurons on the ACC**. **(A) **The representative example of GRP (300 nM) modulation of sIPSCs in a ACC pyramidal neuron. The trace below represents sIPSCs recorded before, during and after GRP application. The bottom 3 traces are presented at an expanded scale. (**B and C**) Time course for the GRP-induced enhancement of sIPSC frequency and amplitude in the neuron shown in (A). Note the effect of GRP is reversible. **(D) **Statistical results showed the GRP-induced enhancement of sIPSC frequency and amplitude in the neuron shown in (A). Note the effect of GRP is reversible. *Indicates a significant difference between control and GRP. * < 0.05, ** < 0.01. **(E) **The facilitatory effect of GRP on sIPSC frequency is concentration dependent (30 nM, n = 6; 300 nM, n = 5; 1000 nM, n = 7). * < 0.05, ** < 0.01, *** < 0.001.

**Figure 5:**
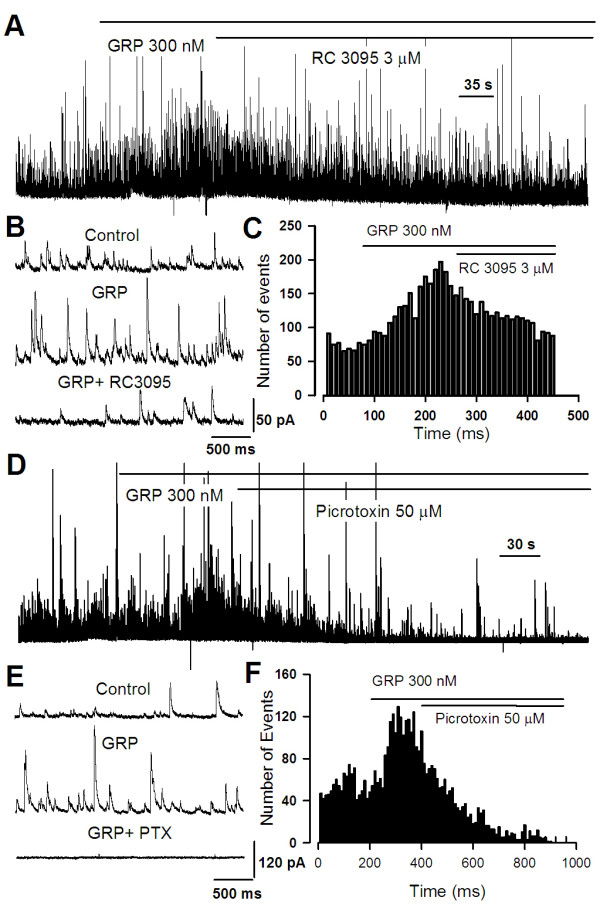
**The GRP-induced sIPSC facilitation is blocked by RC3095 and picrotoxin**. **(A and B) **The effect of GRP (300 nM) could be blocked by RC3095 (3 μM, n = 4), suggesting that the GRP's action is mediated by GRPR (upper). Representative sIPSCs recorded in a pyramidal cell from a control mouse at a holding potential of +10 mV under baseline conditions (upper), during GRP application (middle), and after the GRPR antagonist was added (lower). **(C) **Time course of reversible drug effect of RC3095 (3 μM) to the enhancement of sIPSC induced by GRP treatment. **(D) **The effect of GRP (300 nM) could be blocked by picrotoxin (100 μM, n = 4). Representative sIPSCs recorded in a pyramidal neuron under baseline conditions (upper), during GRP application (middle), and after picrotoxin was added (lower). **(E) **Time course of reversible drug effect of picrotoxin (100 μM) to the enhancement of sIPSC induced by GRP treatment.

Since GRP can excite interneurons, we speculated that activation of GRP receptor would affect the release of GABA in the ACC. To test this idea, we studied the effect of GRP on GABAergic transmission. At a holding potential of 10 mV and with the blockade of NMDA and AMPA/KA current by using AP-5 (50 μM) and CNQX (20 μM), we recorded spontaneous inhibitory postsynaptic currents (sIPSCs) in pyramidal neurons of ACC slices from adult mice. GRP (300 nM) increased both frequency (from 7.0 ± 0.8 Hz to 10.6 ± 1.1 Hz, n = 7, *p *< 0.001) and amplitude (from 17.1 ± 0.5 pA to 20.3 ± 0.6 pA, n = 7, *p *< 0.05) of sIPSCs (Figure 4A-D). This facilitation was reversible after the washout of GRP (Figure 4A-D)). Moreover, the effect of GRP was concentration-dependent. The frequency of sIPSCs was significantly increased to 140.1 ± 7.8% (n = 10, *p *< 0.001) and 183.4 ± 10.6% (n = 10, *p *< 0.001) at 300 and 1000 nM GRP, respectively (Figure 4E). No significant effect was observed for 30 nM GRP (100.5 ± 12.4%, n = 5, *p *= 0.1, Figure 4E). The mean amplitude of sIPSCs was also significantly increased at both 300 nM GRP (110.6 ± 3.9%, n = 5, *p *< 0.05) and 1000 nM GRP (124.8 ± 9.0%, n = 5, *p *< 0.01) (Figure 4E). To further test whether the facilitatory effect of sIPSCs by GRP is mediated by GRP receptor, we applied the selective GRP receptor antagonist RC3095 following the GRP application. We found that RC3095 (3 μM, n = 5) completely eliminate the enhancement of either frequency or amplitude of sIPSCs in the ACC neurons (Figure 5A-C). The application of GABA_A _receptor antagonist picrotoxin (50 μM) abolished all sIPSCs (Figure 5D-F).

**Figure 6:**
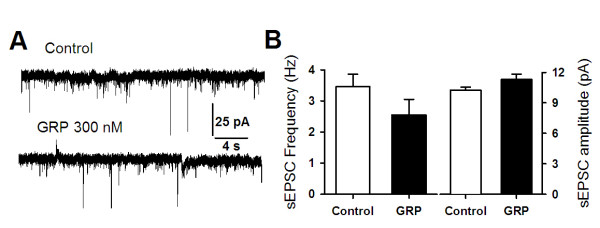
**Activation of GRP receptor by GRP had no effect on sEPSCs in pyramidal neurons of ACC**. **(A) **Typical traces showing the effect of GRP (300 nM) on sEPSCs in a pyramidal neuron. **(B) **Statistical results showed no effect of GRP on either frequency or amplitude of sEPSCs in pyramidal neurons (n = 6).

We next tested if glutamatergic neurotransmission in the ACC was affected by GRP application. At a holding potential of -70 mV and with the blockade of GABA current in the presence of picrotoxin (100 μM), we recorded spontaneous excitatory postsynaptic currents (sEPSCs) from pyramidal neurons of ACC. We found that bath application of GRP (300 nM) had no significant effect on either the frequency or amplitude of sEPSCs in ACC pyramidal neurons (n = 5, Figure 6A-B).

### Increase of GABA release by GRP is action potential dependent

**Figure 7:**
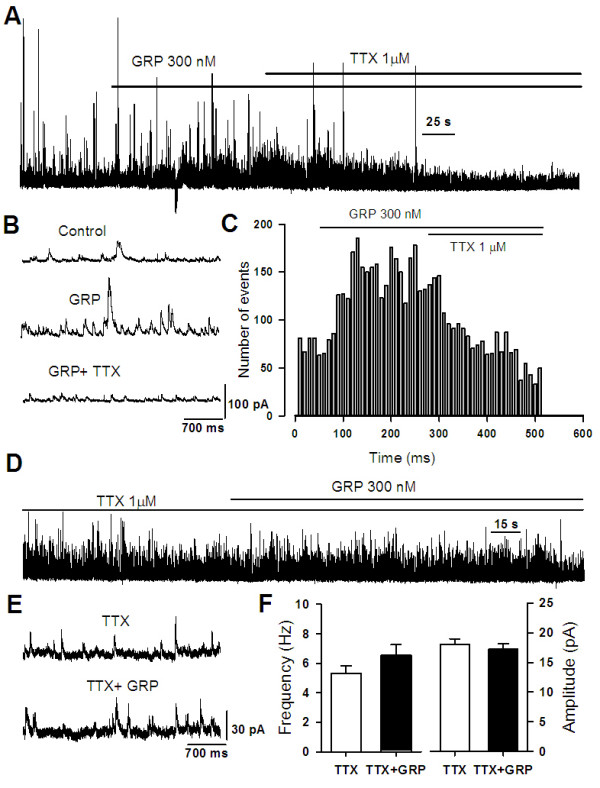
**Increase of GABA release by GRP is action potential dependent**. **(A) **The effect of GRP (300 nM) could be blocked by TTX (1 μM, n = 4). **(B) **Representative sIPSCs recorded in a pyramidal neuron under baseline conditions (upper), during GRP application (middle), and after TTX was added (lower). **(C) **Time course of reversible drug effect of TTX (1 μM) to the enhancement of sIPSC induced by GRP treatment. **(D and E) **Typical examples showing the effect of GRP (300 nM) on mIPSCs in the presence of TTX. Similar results were obtained from an additional 4 neurons. **(F) **Statistical results showed in the presence of TTX (1 μM), GRP did not affect either amplitude or frequency of mIPSCs.

To investigate whether GRP receptors are involved in regulating GABA release in the ACC, we conducted experiments where RC3095 (3 μM) was applied to the bath solution after the GRP application. We found that the facilitatory effect of GRP on sIPSCs was completely reversed (Figure 7A-C, n = 5), suggesting that the effect of GRP is mediated by GRPRs. To further confirm these results, we examined miniature inhibitory postsynaptic currents (mIPSCs) in the presence of TTX (1 μM). Bath application of GRP at three different doses (0.03, 0.3 and 3 μM) did not produce any significant effect on either the frequency or amplitude of mIPSCs (Figure 7D-F). Taken together, these results demonstrate that effects of GRP were not due to a presynaptic mechanism, but rather associated with depolarization and triggering action-potentials in GABAergic interneurons.

### Activation of GRP receptor also increased the frequency of sIPSCs in pyramidal neurons in the basolateral anygdala (BLA) and insular cortex (IC)

**Figure 8:**
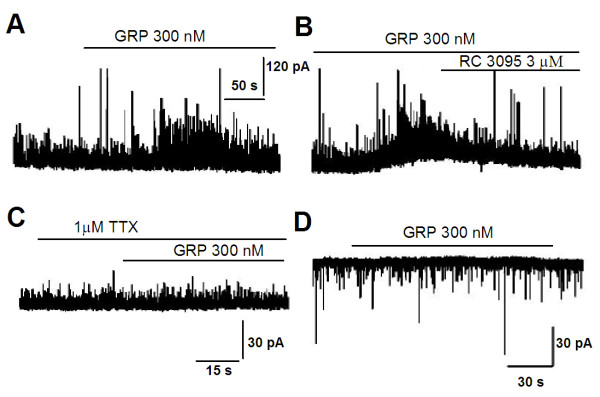
**Activation of GRP receptors increased GABA release in BLA neurons**. **(A) **Bath application of GRP (300 nM) increased the frequency of sIPSCs in pyramidal neurons of BLA (n = 11). **(B) **The effect of GRP was completely blocked by RC 3091 (3 μM) (n = 5). **(C) **No increase of the frequency of sIPSCs was observed in the presence of TTX (1 μM) (n = 5). **(D) **There is no effect of GRP on sEPSCs (n = 4). Holding potential for recording sEPSCs was -70 mV.

**Figure 9:**
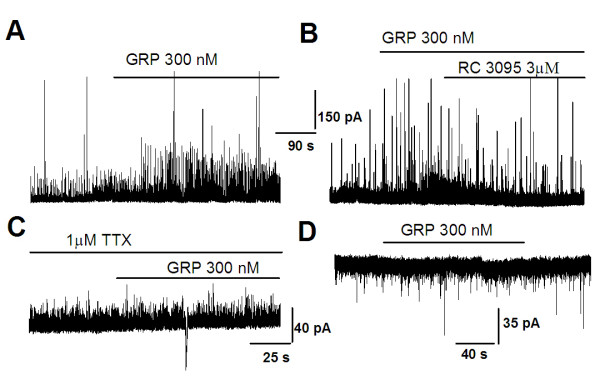
**Activation of GRP receptors increased GABA release in IC neurons**. **(A and B) **Bath application of GRP (300 nM) increased the frequency of sIPSCs in pyramidal neurons of IC (n = 11). **(B) **The effect of GRP was completely blocked by RC 3091 (3 μM) (n = 5). **(C) **However, no increase was observed in the presence of TTX (1 μM) (n = 5). **(D) **There is no effect of GRP on sEPSCs (n = 4). Holding potential for recording sEPSCs was -70 mV.

To examine whether the facilitation of GABAergic transmission by GRP is a general phenomenon in the brain, we tested the effect of GRP on inhibitory transmission in the BLA and IC. Similar to the results found in the ACC, bath application of GRP (300 nM) significantly increase the frequency of sIPSCs recorded in pyramidal neurons in the BLA (*p *< 0.05, n = 8, Figure 8) and IC (*p *< 0.05, n = 8, Figure 9). Consistent with the notion that the facilitatory effects are mediated by the GRP receptor, these facilitation were completely blocked by GRP receptor antagonist, RC3095 (3 μM, n = 6; Figure 8, 9). In the presence of TTX (1 μM), GRP had no effect on mIPSCs (n = 5; Figure 8 and 9), Moreover, GRP had no effect on sEPSCs in either BLA or IC (n = 5, Figure 8 and 9).

## Discussion

In the present study, we used whole-cell patch-clamp recording to study the actions of GRP on ACC neurons in adult mice. Our results provide strong electrophysiological evidence that GRP receptor facilitates inhibitory GABA release in the ACC. Activation of GRP receptor preferentially modulated GABAergic, but not glutamatergic transmission. Furthermore, somatodendritic GRP receptor mediated action potential-dependent GABA release in the ACC occurred in other regions, such as the BLA and IC, indicating a general role of GRP receptor in the modulation of cortical GABAergic transmission.

How GRP facilitate GABAergic transmission and modulate the neuronal circuits in the ACC? Several lines of evidence in the present study suggest that GRP acts on somatodendritic GRP receptor in GABAergic neurons to induce neuronal firing and GABA release in interneurons, thereby decreasing the excitability of pyramidal neurons. Bath application of GRP facilitated sIPSC frequency in a concentration- dependent manner, but GRP had little effect on either frequency or amplitude of mIPSCs. These results are similar to the modulatory effects of the GRP/GRP receptor system on GABAergic transmission in lateral amygdala and hippocampus [18,26]. Interestingly, in the present study, although ACC pyramidal neurons are shown to express GRP receptors, we found little direct effect of GRP on these cell bodies nor on the frequency and amplitude of sEPSC. We cannot completely rule out other possible modulation of GRP on excitatory transmission that cannot be revealed in the present studies. Future studies are clearly needed in further investigating the roles of GRP.

ACC neurons are multi-functional and play important roles in a wide variety of behavioral functions, including sensory pain, memory, emotional and cognitive functions [19-22,27,28]. Glutamate is the fast excitatory transmitter [29] and GABA is the inhibitory transmitter in the ACC [30]. A balance between excitatory and inhibitory transmission is critical for many brain functions. Previous reports show that the GRP in the amygdala is inovolved in behavioral fear [18,31-36]. In addition to the amygdala, recent studies show that lesion of the ACC produced an impairment in trace fear conditioning [37] and electrical stimulation of the ACC induced fear memory [38]. The results of the present study show that GRP may facilitate the GABAergic transmission in the ACC synapses, indicating that GRP may contribute to behavioral fear or trace fear memory by affecting inhibitory transmission within the ACC.

GRP has been implicated in mediation the itch sensation in the spinal cord [14,15]. The possible roles of GRP within the ACC in behavioral itching have not been investigated. Furthermore, a recent work in the spinal dorsal horn suggests that alteration of inhibitory transmission in the spinal cord is important for behavioral itching [39]. It is possible that GRP may also affect spinal inhibitory transmission, in addition to act as a potential transmitter for itch from the periphery. Future studies are clearly needed in the spinal cord. Furthermore, direct evidence for GRP to act as a neurotransmitter for itching is still lacking. In human studies, ACC and IC have been shown to be involved in itch processing [40-45]. Future studies are required to address whether the modulation of GRP in the ACC inhibitory circuit would contribution to itch sensation. In summary, we report here that GRP play an important role in modulating inhibitory transmission within the ACC, IC and amygdala. It is likely that supraspinal GRP may contribute to a wide range of physiological and pathological functions, rather than act as a selective transmitter for itch as reported at the level of spinal cord.

## Conflict of interests

The authors declare that they have no competing interests.

## Authors' contributions

XYC, VM, PL and LJW performed the experiments included in the manuscript. MZ designed the experiments. XYC, LJW and MZ wrote the manuscript. All authors read and approved the final manuscript.
